# Multicenter, International Assessment of the Eighth Edition of the American Joint Committee on Cancer *Cancer Staging Manual* for Conjunctival Melanoma

**DOI:** 10.1001/jamaophthalmol.2019.1640

**Published:** 2019-06-06

**Authors:** Puneet Jain, Paul T. Finger, Bertil Damato, Sarah E. Coupland, Heinrich Heimann, Nihal Kenawy, Niels J. Brouwer, Marina Marinkovic, Sjoerd G. Van Duinen, Jean Pierre Caujolle, Celia Maschi, Stefan Seregard, David Pelayes, Martin Folgar, Yacoub A. Yousef, Hatem Krema, Brenda Gallie, Alberto Calle-Vasquez

**Affiliations:** 1 Department of Ocular Tumor and Orbital Disease, The New York Eye Cancer Center, New York; 2 Ocular Oncology Service, Department of Ophthalmology, University of California, San Francisco; 3 Department of Ophthalmology, Royal Liverpool University Hospital, Liverpool, England; 4 Department of Pathology, Royal Liverpool University Hospital, Liverpool, England; 5 Department of Ophthalmology, Leiden University Medical Center, Leiden, the Netherlands; 6 Department of Ophthalmology, St Roch Hospital, Nice University Hospital, Nice, France; 7 Department of Ophthalmology, St Erik’s Eye Hospital, Karolinska Institute, Stockholm, Sweden; 8 Department of Pathology, St Erik’s Eye Hospital, Karolinska Institute, Stockholm, Sweden; 9 Department of Ophthalmology, Carlos G. Durand Hospital, Buenos Aires, Argentina; 10 The Ocular Oncology Multidisciplinary Clinic, Department of Surgery, King Hussein Cancer Center, Amman, Jordan; 11 The Eye Cancer Clinic, Princess Margaret Cancer Centre, Toronto, Ontario, Canada; 12 Department of Ophthalmology, Calle Ophthalmic and Orbit Center, Bogota, Distrito Capital, Colombia

## Abstract

**Importance:**

Eye cancer staging systems used for standardizing patient care and research need to be validated.

**Objective:**

To evaluate the accuracy of the eighth edition of the American Joint Committee on Cancer (AJCC) *Cancer Staging Manual* in estimating metastatis and mortality rates of conjunctival melanoma.

**Design, Setting, and Participants:**

This international, multicenter, registry-based case series pooled data from 10 ophthalmic oncology centers from 9 countries on 4 continents. A total of 288 patients diagnosed with conjunctival melanoma from January 1, 2001, to December 31, 2013, were studied. Data analysis was performed from July 7, 2018, to September 11, 2018.

**Interventions:**

Treatments included excision biopsy, cryotherapy, topical chemotherapy, radiation therapy, enucleation, and exenteration.

**Main Outcomes and Measures:**

Metastasis rates and 5-year and 10-year Kaplan-Meier mortality rates according to the clinical T categories and subcategories of the eighth edition of the AJCC *Cancer Staging Manual*.

**Results:**

A total of 288 eyes from 288 patients (mean [SD] age, 59.7 [16.8] years; 147 [51.0%] male) with conjunctival melanoma were studied. Clinical primary tumors (cT) were staged at presentation as cT1 in 218 patients (75.7%), cT2 in 34 (11.8%), cT3 in 15 (5.2%), and cTx in 21 (7.3%). There were no T4 tumors. Pathological T categories (pT) were pTis in 43 patients (14.9%), pT1 in 169 (58.7%), pT2 in 33 (11.5%), pT3 in 12 (4.2%), and pTx in 31 (10.8%). Metastasis at presentation was seen in 5 patients (1.7%). Metastasis during follow-up developed in 24 patients (8.5%) after a median time of 4.3 years (interquartile range, 2.9-6.0 years). Of the 288 patients, 29 died (melanoma-related mortality, 10.1%) at a median time of 5.3 years (interquartile range, 1.8-7.0 years). The cumulative rates of mortality among patients with cT1 tumors were 0% at 1 year, 2.5% (95% CI, 0.7%-7.7%) at 5 years, and 15.2% (95% CI, 8.1%-27.4%) at 10 years of follow-up; among patients with cT2 tumors, 0% at 1 year, 28.6% (95% CI, 12.9%-58.4%) at 5 years, and 43.6% (95% CI, 19.6%-77.9%) at 10 years of follow-up; and among patients with cT3 tumors, 21.1% (95% CI, 8.1%-52.7%) at 1 year of follow-up and 31.6% (95% CI, 13.5%-64.9%) at 5 years of follow-up. Patients with cT2 and cT3 tumors had a significantly higher cumulative mortality rate compared with those presenting with cT1 tumors (log-rank *P* < .001). Patients with ulcerated melanomas had significantly higher risk of mortality (hazard ratio, 7.58; 95% CI, 1.02-56.32; *P* = .04).

**Conclusions and Relevance:**

This multicenter, international, collaborative study yielded evidence that the conjunctival melanoma staging system in the eighth edition of the AJCC *Cancer Staging Manual* can be used to accurately estimate metastasis and mortality rates. These findings appear to support the use of AJCC staging as a tool for patient care and research.

## Key Points

**Question:**

Can the eighth edition of the American Joint Committee on Cancer (AJCC) *Cancer Staging Manual* for conjunctival melanoma be used to accurately estimate metastasis and mortality rates?

**Findings:**

In this case series of 288 patients, there was a significantly higher cumulative mortality rate among patients with cT2 and cT3 conjunctival melanoma compared with those presenting with cT1 conjunctival melanoma.

**Meaning:**

Higher T-staged tumors were associated with both an earlier and greater incidence of metastasis; therefore, the results from this multicenter, international registry study support the use of the eighth edition AJCC staging system for conjunctival melanoma.

## Teaser

This case series assesses whether the eighth edition of the American Joint Committee on Cancer (AJCC) *Cancer Staging Manual* can be used to accurately estimate mortality rates of conjunctival melanoma.

## Introduction

Conjunctival melanomas comprise 2% of all ocular tumors and 5% of eye melanomas^1,2,3^; however, their incidence is increasing.^4^ Tumor-related mortality is reported to be 30%.^5^ Reliable prognostic tools are needed to enhance clinical and basic science research.

Published by the American Joint Committee on Cancer (AJCC) and used by the Union for International Cancer Control, the TNM staging system is widely implemented around the world. In 2016, the AJCC Ophthalmic Oncology Task Force created the eighth edition of the conjunctival melanoma staging system with data from more than 50 eye cancer specialists in 18 countries. Changes from the seventh edition include further defining the tumors’ circumferential extent to define the clinical T categories.^6^

Widely accepted by medical oncology, ophthalmic oncology, radiation oncology, and medical journals, the AJCC-TNM staging system serves to standardize data reporting, case-to-case prognosis, and selection of the most suitable treatment for conjunctival melanoma. Specialists who stage cancers have recognized the need for standardized collaborative data sharing.^7,8^ We performed, to our knowledge, the first multicenter, international study to evaluate the accuracy of the eighth edition of the AJCC *Cancer Staging Manual* in estimating mortality rates of metastasis from conjunctival melanoma.

## Methods

Patients diagnosed with conjunctival melanoma from January 1, 2001, to December 31, 2013, were included in this multicenter, international study. Data were collected retrospectively and entered into a secure online database. Data analysis was performed from July 7, 2018, to September 11, 2018. This study adhered to the tenets of the Declaration of Helsinki^9^ and the Health Insurance Portability and Accountability Act of 1996. All participating centers obtained internal institutional review board approval to perform retrospective medical record reviews and contribute data to the AJCC Ophthalmic Oncology Task Force Conjunctival Melanoma Registry. The Princess Margaret Cancer Centre determined that individual patient consent was not required because there were no patient identifiers.

### Conjunctival Melanoma Registry Centers

In 2015, at the first Eye Cancer Working Day, held at the Curie Institute (Paris, France), 158 eye cancer specialists were invited to participate in this conjunctival melanoma registry. Conceptualized as an internet-based data-sharing registry, the data fields were designed by participating eye cancer specialists. The resulting registry included data from 10 ophthalmic oncology centers in 9 countries (2 in the United States and 1 each in Canada, Colombia, Argentina, France, Netherlands, United Kingdom, Sweden, and Jordan) on 4 continents (North America, South America, Europe, and Asia).

### Data Security

The following features were incorporated to ensure data security and study participant privacy. There were no personal identifiers. The participants were assigned a unique identification number at their local institution that linked patients to locally stored corresponding identification numbers to ensure accuracy of follow-up and outcomes. Secure Socket Layer encryption was used to prevent information from being viewed by third parties. Registry access was limited to participants with user accounts. Therefore, the login page required a unique username and password combination to access the application. The database incorporated record locking to prevent different users from accessing the same record for the same study participant at the same time. This locking prevented unintentional overwriting or data corruption. In addition, audit trails were automatically created. Such logs were stored in a separate, secured directory and were not available online.

### Tumor Staging

Clinical (cT) and pathologic (pT) staging were performed according to the staging system for conjunctival melanoma in the eighth edition of the AJCC *Cancer Staging Manual*^6^ (Table 1). According to the AJCC, T1 disease is confined to the bulbar conjunctiva, T2 disease affects the nonbulbar conjunctiva and/or caruncle, and local invasion to adjacent tissues elevates the conjunctival melanoma to T3 disease. T4 disease denotes central nervous system invasion.^6^

**Table 1.** Conjunctival Melanoma as Defined by the Eighth Edition of the American Joint Committee on Cancer* Cancer Staging Manual*

Category	Criteria
**Clinical Tumor (cT)**	
TX	Primary tumor cannot be assessed
T0	No evidence of primary tumor
T1	Tumor of the bulbar conjunctiva
T1a	<1 Quadrant
T1b	≥1 to <2 Quadrants
T1c	≥2 to <3 Quadrants
T1d	≥3 Quadrants
T2	Tumor of the nonbulbar (forniceal, palpebral, and tarsal) conjunctiva and tumor involving the caruncle
T2a	Noncaruncular and ≤1 quadrant of the nonbulbar conjunctiva involved
T2b	Noncaruncular and >1 quadrant of the nonbulbar conjunctiva involved
T2c	Caruncular and ≤1 quadrant of the nonbulbar conjunctiva involved
T2d	Caruncular and >1 quadrant of the nonbulbar conjunctiva involved
T3	Tumor of any size with local invasion
T3a	Globe
T3b	Eyelid
T3c	Orbit
T3d	Nasolacrimal duct and/or lacrimal sac and/or paranasal sinuses
T4	Tumor of any size with invasion of the central nervous system
**Pathologic Tumor (pT)**	
TX	Primary tumor cannot be assessed
T0	No evidence of primary tumor
Tis	Melanoma confined to conjunctival epithelium
T1a	Tumor of the bulbar conjunctiva with invasion of the substantia propria, not >2.0 mm in thickness
T1b	Tumor of the bulbar conjunctiva with invasion of the substantia propria, >2.0 mm in thickness
T2a	Tumor of the nonbulbar conjunctiva involved with invasion of the substantia propria not >2.0 mm in thickness
T2b	Tumor of the nonbulbar conjunctiva with invasion of the substantia propria >2.0 mm in thickness
T3a	Globe
T3b	Eyelid
T3c	Orbit
T3d	Nasolacrimal duct, lacrimal sac, and/or paranasal sinuses
T4	Tumor of any size with invasion of the central nervous system
**Regional Lymph Node (N)**	
NX	Regional lymph nodes cannot be assessed
N0	No regional lymph node metastasis
N1	Regional lymph node metastasis
**Distant Metastasis (M)**	
M0	No distant metastasis
M1	Distant metastasis

### Definitions

Ophthalmic oncology examinations were performed according to the standards and practice of each participating center. These practices included slitlamp and gonioscopic photography and high-frequency anterior segment ultrasonography to rule out intra-ocular invasion. Metastases were subgrouped at presentation or during follow-up. Systemic screening for metastasis was performed according to the custom and practice of the local institutions. Time to death was defined as the interval between date of diagnosis and the date of metastasis. Because at the time of the study no curative treatment for metastatic conjunctival melanoma existed, metastasis and mortality were deemed to be equivalent for the study.

### Statistical Analysis

Means (SDs) or medians (interquartile ranges [IQRs]) were used to express continuous variables, whereas categorical variables were expressed as proportions. Group comparisons were made using the *t* test or Wilcoxon rank sum test for nonparametric variables. Analysis of variance or the Kruskal-Wallis test was used to compare variables across more than 2 groups. The χ^2^ test or Fisher exact test was used to analyze group differences across categorical variables. Separate survival analyses were performed using metastasis and mortality as the censoring variable, and Kaplan-Meier curves were plotted to depict cumulative survival rates at various time points.

Because of the number of patients analyzed in this study, the different tumor subgroups were combined into T1, T2, and T3 for plotting meaningful Kaplan-Meier curves. Comparison between the survival rates of different subgroups was analyzed using the log-rank test. The survival probability for each outcome was assessed using Cox proportional hazards regression models and displayed using hazard ratios (HRs). Covariates used for adjusting HRs were those with a *P* < .10 in univariate models and those that were associated with mortality in previous studies^3,5,7^ (tumor location, tumor invasion, ulceration, and lymphatic invasion).

Data were exported into Microsoft Excel (Microsoft Corp) for analysis. Data were analyzed using Stata, version 12.1 (StataCorp) statistical analysis software package, and a 2-tailed *P* < .05 was considered to be statistically significant.

## Results

### Demographics

A total of 288 eyes from 288 patients (mean [SD] age, 59.7 [16.8] years; range, 15-95 years; 147 [51.0%] male) with conjunctival melanoma were studied. All patients had the relevant data set; therefore, no patient was excluded from the study. There was slight preponderance of right eye involvement (n = 154 [53.4%]). The median follow-up time (from the time of definitive treatment) was 4.4 years (IQR, 2.3-6.9 years; range, 1 month to 14.3 years).

### Clinical Staging

Table 1 gives the AJCC definitions of tumor size, extent, and pathologic characteristics of conjunctival melanoma. On the basis of the eighth edition of the AJCC *Cancer Staging Manual*, the clinical T categories were cT1 in 218 patients (75.7%), followed by cT2 (34 [11.8%]), cT3 (15 [5.2%[), and cTx (21 [7.3%]). A detailed analysis of subgroups found 117 eyes (40.6%) with T1a, 63 (21.9%) with T1b, 8 (2.8%) with T1c, 2 (0.7%) with T1d, 28 (9.7%) with unspecified T1 disease, 8 (2.8%) with T2a, 5 (1.7%) with T2b, 8 (2.8%) with T2c, 8 (2.8%) with T2d, 5 (1.7%) with unspecified T2 disease, 1 (0.7%) with T3a, 10 (3.5%) with T3b, and 4 (1.4%) with T3c disease (Table 2). There were no cT4 tumors.

**Table 2.** Cumulative Mortality Rates Based on American Joint Committee on Cancer Stage Over Time

Category	No. of Patients	Melanoma-Related Deaths, No. (%)	Cumulative Mortality, % (95% CI)		
			1 y	5 y	10 y
**Clinical T Category**^a^					
Tx	21	3 (14.3)	0	9.1 (1.3-49.1)	NA
T1a	117	9 (7.7)	0	3.5 (0.8-13.3)	20.5 (9.8-40.3)
T1b	63	3 (4.8)	0	1.9 (0.3-13.1)	10.1 (2.1-42.3)
T1c	8	1 (12.5)	0	0	50.0 (9.0-99.4)
T1d	2	0	0	NA	NA
T2a	8	0	0	0	NA
T2b	5	2 (40.0)	0	60.0 (17.1-98.8)	NA
T2c	8	0	0	0	NA
T2d	8	1 (12.5)	0	0	NA
T3a	1	1 (100)	0	NA	NA
T3b	10	2 (20.0)	11.1 (1.6-56.7)	11.1 (1.6-56.7)	NA
T3c	4	4 (100)	33.3 (5.5-94.6)	66.7 (22.6-99.1)	NA
**Pathologic T Category**					
Tx	31	3 (9.7)	0	0	NA
Tis	43	0	0	0	0
T1a	123	7 (5.7)	0	2.5 (0.6-9.7)	13.9 (6.1-29.9)
T1b	46	3 (6.5)	0	5.0 (0.7-30.5)	5.0 (0.7-30.5)
T2a	12	5 (41.7)	0	32.7 (11.5-72.3)	77.5 (38.5-98.9)
T2b	21	4 (19.0)	0	24.2 (6.3-69.5)	43.2 (15.2-85.5)
T3a	1	1 (100)	NA	NA	NA
T3b	6	2 (33.3)	20.0 (3.0-79.6)	NA	NA
T3c	5	4 (80.0)	25.0 (3.9-87.2)	50.0 (15.5-94.2)	NA

Abbreviation: NA, not applicable.

^a^ Unspecified cT1 disease in 28 (9.7%) and unspecified cT2 disease in 5 (1.7%).

### Pathologic Staging

Pathologic T categories according to the eighth edition of the AJCC *Cancer Staging Manual* were pTis in 43 (14.9%), pT1 in 169 (58.7%), pT2 in 33 (11.5%), pT3 in 12 (4.2%), and pTx in 31 (10.8%). Subgroup details were as follows: T1a in 123 (42.7%), T1b in 46 (16.0%), T2a in 12 (4.2%), T2b in 21 (7.3%), T3a in 1 (<0.7%), T3b in 6 (2.1%), and T3c in 5 (1.7%) (Table 2).

### Nodes

Nodal status at presentation was N0 in 209 (72.6%) followed by N1 in 13 (4.5%) and Nx in 66 (22.9%). Ipsilateral preauricular and cervical lymph nodes were reported to be involved in 9 of 13 patients (69.2%), and 4 were uncategorized.

### Metastasis

Metastasis classification at presentation was M0 in 216 (75%) and M1 in 5 (1.7%); for 67 patients (23.3%), these data were not available. Of the 5 patients with metastasis, 3 had pulmonary metastasis (1 of whom had synchronous bone involvement) and 2 had hepatic metastasis. An additional 24 of 283 patients (8.5%) developed metastasis during follow-up (median time, 4.3 years; IQR, 2.9-6.0 years). The liver was the most common site of metastasis, found in 11 patients (45.8%), followed by the lungs in 9 patients (37.5%), brain in 3 (12.5%), bone in 2 (8.3%), and abdomen in 2 (8.3%), with 1 case each in the bladder, peritoneum, parotid gland, and skin. Multiorgan metastasis was seen in 10 of the 24 patients (41.7%), and sites of metastasis were not documented in 3 patients (12.5%).

### Cumulative Mortality Rates According to Clinical Stage

**Figure 1.**
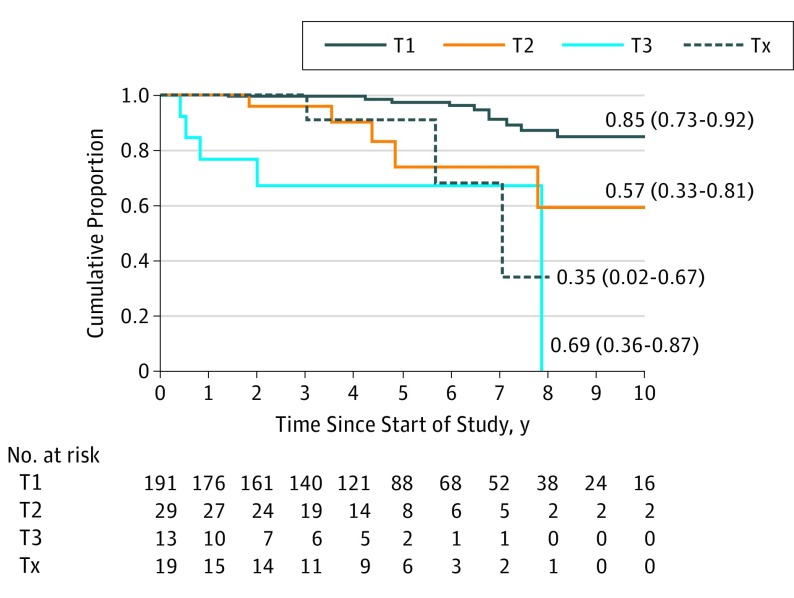
Kaplan-Meier Survival Curves Showing Cumulative Survival Rates for Patients With Different Clinical T Staging Cumulative survival rates are shown as metastasis-free point estimates. HR indicates hazard ratio.

Of the 288 patients, 29 (10.1%) died of conjunctival melanoma at a median time of 5.3 years after diagnosis (IQR, 1.8-7.0 years). The cumulative mortality rates were 0% at 1 year, 2.5% (95% CI, 0.7%-7.7%) at 5 years, and 15.2% (95% CI, 8.1%-27.4%) at 10 years of follow-up for patients with cT1 stage disease; 0% at 1 year, 28.6% (95% CI, 12.9%-58.4%) at 5 years, and 43.6% (95% CI, 19.6%-77.9%) at 10 years of follow-up for cT2 disease; and 21.1% (95% CI, 8.1%-52.7%) at 1 year and 31.6% (95% CI, 13.5%-64.9%) at 5 years of follow-up for cT3 disease, indicating that patients with cT2- and cT3-staged conjunctival melanomas had a significantly higher cumulative mortality rate compared with those with cT1 disease (log-rank *P* < .001), but the mortality rates among cT2, cT3, and Tx were not significantly different (log-rank *P* = .13). Cumulative mortality rates of subgroups of cT1, cT2, and cT3 disease are given in Table 2. The Kaplan-Meier comparative survival curves for cT1, cT2, and cT3 disease are shown in Figure 1.

### Cumulative Mortality Rates According to Pathologic Stage

**Figure 2.**
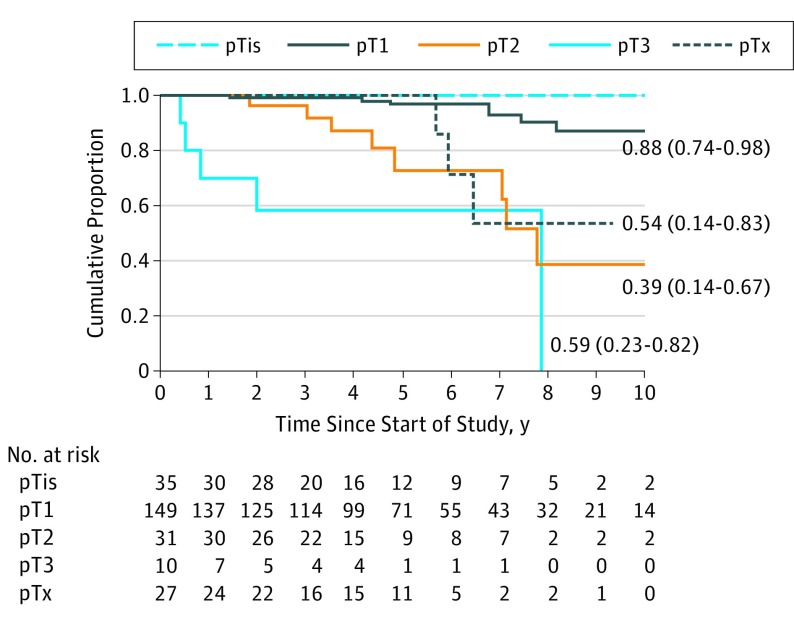
Kaplan-Meier Survival Curves Showing Cumulative Survival Rates for Patients With Different Pathological T Staging Cumulative survival rates are shown as metastasis-free point estimates. HR indicates hazard ratio.

The cumulative mortality rates with respect to pathologic categories and at various time points were 0% at 1 year, 3.1% (95% CI, 0.9%-9.5%) at 5 years, and 13.6% (95% CI, 6.1%-26.4%) at 10 years of follow-up for eyes with pT1 conjunctival melanoma; 0% at 1 year, 27.2% (95% CI, 11.7%-55.6%) at 5 years, and 61.0% (95% CI, 33.4%-89.8%) at 10 years of follow-up for pT2; and 30.0% (95% CI, 10.8%-67.1%) at 1 year and 41.7% (95% CI, 17.9%-77.1%) at 5 years for pT3 (Table 2), indicating that patients with pT2 and pT3 tumors had a significantly higher cumulative mortality rate compared with those with pT1 (log rank *P* < .001), but the mortality rate for pT2 was slightly lower than that for pT3 (log rank *P* = .06). No differences were found for other pairwise group comparisons. The Kaplan-Meier comparative survival curves for pT1, pT2, and pT3 are shown in Figure 2.

On multivariable Cox proportional hazards regression analysis, patients with higher cT stages (cT2, cT3) had 3 times greater risk of mortality (HR, 3.25; 95% CI, 1.48-7.11; *P* = .003) compared with those with cT1. Patients with conjunctival melanomas reported to have ulceration at presentation also had a significantly higher risk of mortality (hazard ratio, 7.58; 95% CI, 1.02-56.32; *P* = .04) (Table 3). This study also notes that involvement of the plica, caruncle, and lymphatic invasion likely did not influence mortality rates. Although caruncular and plical involvement were not a significant risk factor for metastasis, there were 15 T3-staged tumors (4.5%).^10^

**Table 3.** Cox Proportional Hazards Regression Model for Variables Associated With Metastasis Mortality

Variable	Patients, No. (%) (N = 288)	Reference	Hazard Ratio (95% CI)	
			Univariate Analysis	Multivariable Analysis
Age	NA	1-y Increment	1.01 (0.98-1.04)	NA
Male	147 (51.0)	Female	0.82 (0.36-1.89)	NA
Ulceration (n = 143)	14 (9.8)	No ulceration	7.01 (1.77-27.61)	7.58 (1.02-56.32)^a^
Plica involved (n = 287)	31 (10.8)	No involvement	1.38 (0.88-2.15)	NA
Caruncle involved (n = 288)	32 (11.1)	No involvement	1.33 (0.86-2.05)	NA
Tumor stage				
T2	34 (11.8)	T1	4.59 (1.56-13.52)	5.49 (0.35-8.62)
T3	15 (5.2)	T1	17.44 (5.78-52.58)	11.64 (1.78-17.22)^b^
Tx	21 (7.3)	T1	5.44 (1.54-21.32)	3.51 (0.44-4.92)
Invasive melanoma^a^	219 (76.0)	No invasion	2.35 (1.12-4.96)	0.96 (0.29-2.95)
Tumor thickness (n = 201)	NA	1-mm Increment	1.20 (1.04-1.49)	0.94 (0.55-1.53)
Lymphatic invasion (n = 148)	10 (6.8)	No invasion	7.49 (1.49-37.51)	7.48 (0.40-15.05)

Abbreviation: NA, not applicable.

^a^ *P* = .04.

^b^ *P* = .008.

## Discussion

Multicenter, international tumor registries are providing new medical evidence by allowing analysis of large numbers of rare tumors.^2,5,7,8,11,12,13,14^ This registry provided evidence from a large case series of eyes with conjunctival melanoma. The findings suggest that the eighth edition of AJCC *Cancer Staging Manual* for conjunctival melanoma is valid. Specifically, the rates of 1-, 5-, and 10-year mortality increased with the advancing initial cT stage of the primary tumor. The study also revealed independent factors associated with increased mortality, which included tumor thickness, tumor invasion, and ulceration (Table 3). Caruncular or plical involvement did not affect mortality.

The finding that the cumulative rate of mortality with respect to clinical categories showed a steep increase from T1 (3%) to T2 (28%) at the 5-year follow-up suggests the validity of the staging system. This increase in mortality supports the importance of tumor location, with nonbulbar tumors (T2) tending to be more aggressive or harder to control than bulbar tumors (T1). The cumulative 10-year mortality was 3-fold greater for T2 disease compared with T1 disease (43.6% vs 15.2%). Patients with T3 disease were the only ones with mortality at 1 year (cumulative mortality rate of 21%). The 5-year cumulative mortality rates were comparable for patients with T2 and T3 tumors. However, on subgroup analysis, the number of patients in the T3 group was insufficient for 5-year analysis. Although there are no multicenter data on cumulative mortality rates to date, previous single-center studies^2,3,5,7,13,14^ have suggested that larger tumors are associated with a higher incidence of metastasis and mortality. All patients in whom primary tumor could not be assessed (ie, cTx and pTx categories) were censored for the calculation of mortality rates.

In this study, the cumulative mortality rates were comparable for both clinical and pathologic T categories, which suggests that clinical staging is at least as useful as pathologic staging for mortality. Univariate and multivariable HR analyses revealed trends that were comparable to previous studies (Table 3).^2,7,10,13,14,15,16,17^ However, contrary to the AJCC definitions and prior studies,^10,12,15^ our data analysis did not support conjunctival melanoma involvement of the plica and caruncle being associated with higher mortality rates. A small case series suggested that, like other mucous membrane melanomas, immunotherapy may be effective in the treatment of advanced local and metastatic conjunctival melanoma.^18,19^

This study supports the continued use of the conjunctival melanoma staging system published in the eighth edition of the AJCC *Cancer Staging Manual* because use of the tool helped to accurately estimate melanoma-related mortality. The study suggests a potential for modification of weighting of caruncular and plical involvement as well as T4 definitions. Universal staging allowed for large multicenter, international cooperation investigating a rare ophthalmic cancer. This study provided data analysis to improve our knowledge of conjunctival melanoma.

### Limitations

The main limitation of this study is its retrospective design. Another limitation is the small number of patients in some cT categories; 75.7% of the tumors were cT1 staged, and there were no cT4 tumors. Because there were no criteria for case selection, this distribution represents the clinical experience of the multiple international centers involved in this study. Ethnic/racial backgrounds were not collected as data; however, because the data were derived from 4 continents, the registry included a broad spectrum of patients. In contrast, Zhou et al^13^ reported on mostly cT2 and cT3 tumors from the Chinese population.^5,7^

## Conclusions

These results suggest that the conjunctival melanoma staging system in the eighth edition of AJCC *Cancer Staging Manual* can be used to accurately estimate mortality related to metastatic disease. The findings also suggest that international, multicenter, registry-based studies of rare cancers can be performed using internet-based data sharing.
